# Neonatal Impedance Cardiography in Asphyxiated Piglets—A Feasibility Study

**DOI:** 10.3389/fped.2022.804353

**Published:** 2022-02-25

**Authors:** Gazmend Berisha, Rønnaug Solberg, Claus Klingenberg, Anne Lee Solevåg

**Affiliations:** ^1^Department of Paediatric and Adolescent Medicine, Akershus University Hospital, Lørenskog, Norway; ^2^Institute of Clinical Medicine, University of Oslo, Oslo, Norway; ^3^Department of Pediatric Research, Institute of Surgical Research, Oslo University Hospital Rikshospitalet, University of Oslo, Oslo, Norway; ^4^Department of Pediatrics, Vestfold Hospital Trust, Tønsberg, Norway; ^5^Department of Paediatrics, University Hospital of North Norway, Tromsø, Norway; ^6^Paediatric Research Group, Department of Clinical Medicine, UiT, The Arctic University of Norway, Tromsø, Norway; ^7^Department of Paediatric and Adolescent Medicine, Oslo University Hospital, Nydalen, Norway

**Keywords:** asphyxia neonatorum, animal model, cardio-circulatory monitoring of transition, bioimpedance, hemodynamics

## Abstract

**Objectives:**

Impedance cardiography (ICG) is a non-invasive method for continuous cardiac output measurement and has the potential to improve monitoring and treatment of sick neonates. PhysioFlow^®^ is a signal-morphology ICG-system showing promising results in adults with low and high cardiac output, but no data from neonates or neonatal models exist. The aim of this study was to investigate PhysioFlow^®^ feasibility in asphyxiated newborn piglets.

**Methods:**

Fifteen piglets, under continuous arterial heart rate (HR) and blood pressure (BP) monitoring, were asphyxiated until asystole. Cardiopulmonary resuscitation was performed and the piglets monitored after return of spontaneous circulation (ROSC). Arterial lactate was measured at baseline, every 5 min throughout asphyxiation, at asystole, and at 10 min and later every 30 min after ROSC. PhysioFlow^®^ measured cardiac stroke volume (SV) and HR, and calculated cardiac index (CI) (L/m^2^/min). Registrations with a signal quality < 75% were excluded, and registrations recorded for 30 min from start of asphyxia analyzed. Pearson correlations were calculated for CI; and HR, mean BP and blood lactate.

**Results:**

The piglets were asphyxiated for median (interquartile range) 30 (20–35) min and had a lactate at asystole of 15.0 (9.1–17.0) mmol/L. Out of a total of 20.991 registrations in all animals combined, there were 10.148 (48.3%) registrations with a signal quality ≥ 75%. Signal quality ≥ 75% varied in individual piglets from 7 to 82% of registrations. We analyzed 1.254 registrations recorded 30 min from initiation of asphyxia, i.e., in piglets with brief asphyxia times, this included cardiopulmonary resuscitation and post-ROSC observation. There was a positive correlation between CI and SVI (*r* = 0.90, *p* < 0.001), and between CI and HR (*r* = 0.446, *p* < 0.001). There was no correlation between CI, or mean BP or lactate (*p* = 0.98 and 0.51, respectively).

**Conclusion:**

About half of ICG-registrations in asphyxiated piglets were of good quality. However, signal quality was highly variable between piglets. In total, there was a higher proportion of reliable ICG-registrations than reported from clinical delivery room studies using electrical velocimetry. Our data are physiologically plausible and supports further research evaluating PhysioFlow^®^ for cardiac output monitoring in perinatal asphyxia. In particular, factors influencing inter-individual variations in signal quality should be explored.

## Introduction

Poor tissue oxygenation and perfusion in critically ill infants can lead to failure of vital organs including the brain, heart, lungs and liver. However, the assessment of tissue oxygenation and perfusion in these infants is challenging, and often relies on indirect methods including pulse oximetry, heart rate (HR) and blood pressure (BP) monitoring. Cardiac output (CO) may more accurately reflect the hemodynamic situation compared with e.g., BP (1) and can be used as an indirect measure of systemic blood flow and perfusion (2). Observational studies have shown poor correlation between BP and CO (2), and BP alone provides limited information about tissue perfusion. Lactic acidosis is a common feature of perinatal asphyxia, with prolonged lactic acidosis being associated with a higher mortality (3, 4). CO may be compromised during induced hypothermia therapy of asphyxiated infants (5), and elevated lactate has been suggested to be used as a surrogate marker of suboptimal hemodynamic status in asphyxiated newborns treated with hypothermia (6).

Doppler echocardiography is a non-invasive method of evaluating CO. The formula stroke volume (SV) x HR is used to provide non-invasive and intermittent CO-values. Transesophageal echocardiography can be used for continuous trend monitoring (7), but current Doppler probes are too large for use in newborns. There are other methods for continuously measuring and monitoring CO (8). Most of these methods are invasive or expensive and/or have limited applicability for other reasons. Thermodilution through a pulmonary artery catheter is used in intensive care contexts (9) and is considered the gold standard. In addition to being invasive, the method requires highly specialized expertise and is not routinely used in newborn infants. Pulse contour analysis is another Doppler-based method that measures SV and provides a continuous CO estimation (10). However, the method has not proven to be reliable in children.

The term impedance cardiography (ICG) is used synonymously with thoracic electrical bioimpedance and electrical impedance plethysmography. Disposable sensors on the neck and chest/torso are used to register electrical (electrical conductivity) and impedance changes in the chest throughout the cardiac cycle, which are used to measure and calculate hemodynamic parameters continuously and non-invasively. The method was first reported in the 1960s (8), but did not come into widespread use until the 1980s (11). It has received increased attention over the last decade as a result of technological advances and improved agreement with invasive CO measurements. The method is easily applicable and is relatively inexpensive to buy and use. These features make ICG potentially attractive for monitoring of supportive therapy including fluid and vasoactive drugs. The method is increasingly used in adults, but is so far little used in newborns.

Some ICG devices process the impedance signal based on how red blood cells are oriented in cardiac systole (electrical velocimetry = EV). EV contrasts to classical bioimpedance, which analyzes volumetric changes in the aorta to determine SV. Although one study showed a good correlation between EV and echocardiography CO (12), EV has limitations including a high sensitivity to noise and low validity in high- and low CO states. In an effort to resolve these issues, signal-morphology ICG (SM-ICG) and bioreactance ICG (BR-ICG) methods have been developed. SM-ICG and BR-ICG utilize two different types of ICG signal filters, and SM-ICG is claimed to apply “a more advanced filtering technology with proven results in the most difficult measurement environments” (13). The PhysioFlow^®^ (Manatec Biomedical, Folschviller, France) SM-ICG has shown promising results in adults without structural heart disease in various clinical settings using direct Fick and echocardiographic comparisons (14–17). However, a recently published study of 23 patients 0–8 years [median body surface area 0.54 m^2^ (range, 0.21–1.00 m^2^)] where most had congenital heart disease showed limitations when comparing Physioflow^®^ with phase contrast magnetic resonance imaging (MRI) CO (18). There is no published data on Physioflow^®^ used in asphyxia, and no data in newborn infants or models exist. Thus, the aim of this study was to investigate the feasibility of PhysioFlow^®^ in asphyxiated newborn piglets with both low- and high-cardiac output during and after asphyxiation and cardiopulmonary resuscitation.

## Materials and Methods

The animals in this study were part of a randomized controlled trial investigating brain mitochondrial complex activity after asphyxia and cardiopulmonary resuscitation (not published). The experimental protocol has been described previously (19), except for the post-resuscitation observation period, which was longer in the present study (up until 9.5 h vs. previously 4 h).

### Experimental Protocol

Newborn piglets have similar anatomy and pathophysiology to newborn infants at near-term gestation. Animals arrived at our facility on the same day as the experiments were conducted. As these were acute experiments, there was no need for housing of the animals. The animals were handled in accordance with the European Guidelines for the use of experimental animals by researchers certified by the Federation of European Laboratory Animals Science Association (20).

Fifteen (ten female) piglets 12–36 h old were sedated, intubated, mechanically ventilated and asphyxiated until asystole, as previously described (19). Briefly, after surgical instrumentation, the piglets were stabilized for at least 1 h before the ventilator FiO_2_ was reduced to 0.08, and the ventilator rate reduced every 10 min until asystole occurred. Asystole was defined as no audible heart beat (stethoscope) and loss of arterial line pulsatility. Arterial lactate was measured with a Blood Gas Analyzer 860 (Ciba Corning Diagnostics, Midfield, MA, USA) at baseline, every 5 min throughout asphyxiation and at asystole, and then at 10 min and later every 30 min after return of spontaneous circulation (ROSC). Arterial BP was continuously monitored through a catheter in the left common carotid artery and manually registered at the same time points as the lactate measurements. Piglets achieving ROSC, were euthanized at predetermined time points as dictated by the mitochondrial complex activity protocol, at a maximum of 9.5 h after ROSC. Piglets not achieving ROSC were euthanized or died at asystole (no resuscitation/no ROSC).

**Figure 1:**
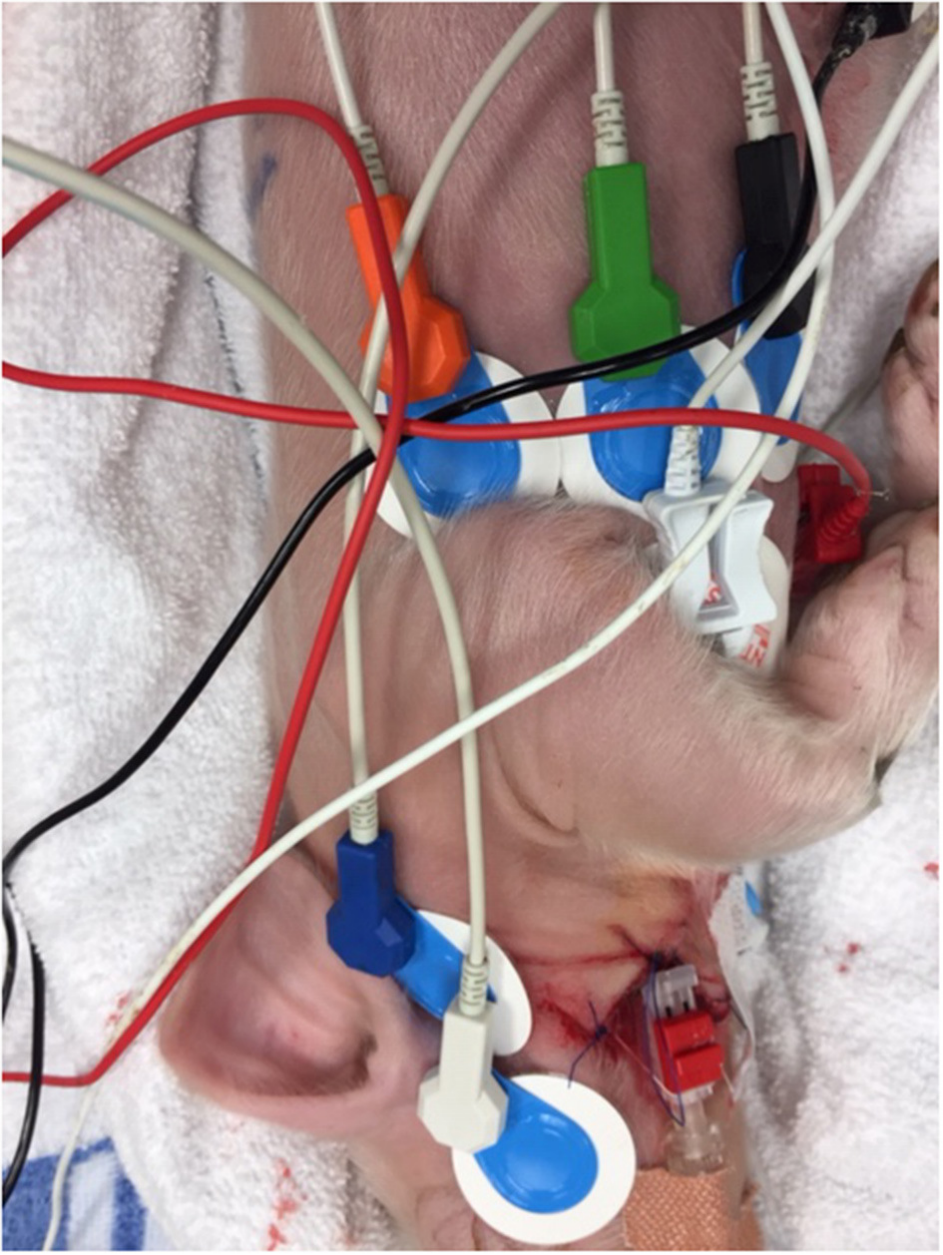
The PhysioFlow^®^ uses 6 snap button electrodes: 2 electrodes on the neck, 2 at the xiphisternum, and 1 on each side of the chest. The blue electrodes are the PhysioFlow^®^ electrodes.

**Figure 2:**
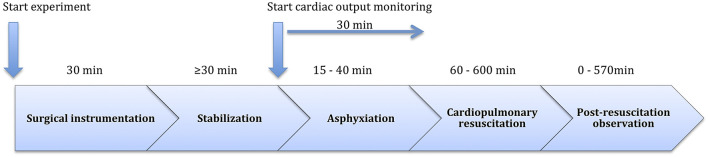
Graphical presentation of the study time line.

For ICG monitoring, according to manufacturer recommendations, six electrodes (Ambu^®^ blue, Ballerup, Denmark) were used: 2 on the neck, 2 at the xiphisternum, and 1 on each side of the chest (Figure 1). PhysioFlow^®^ measures cardiac SV and HR, and calculates cardiac output (CO) (l//min). For all piglets, we used an estimated body surface area of 0.14 m^2^ to calculate cardiac index (CI), which was the main outcome parameter of the study. After a 20 s calibration, the CI (averaged over 10 cycles) was continuously obtained and downloaded to a notebook computer. Timik AS (Norway) provided us with a PhysioFlow^®^ device with program ware version 2.7.4 and a built-in proprietary artifact detection algorithm which is not different from previous generations. Adequate signal quality was detected by a color graph and a percentage signal quality. For research purposes it is essential to be strict with signal quality. According to the manufacturer of PhysioFlow^®^, signal quality ≥ 75% indicates reliable results for clinical use (Timik AS, Norway, personal communication). Thus, we excluded registrations with a signal quality < 75%. Due to different observation times for individual piglets as dictated by protocol, for the purpose of standardization, we analyzed registrations made 30 min from initiation of asphyxia, which included resuscitation and post-resuscitation observation in the piglets where asystole occurred after <30 min of asphyxia (Figure 2).

### Approvals

The Norwegian Council for Animal Research approved the experimental protocol (approval number 17447). The study is presented according to the ARRIVE guidelines (21) (checklist provided as Supplementary Material).

### Data Handling and Statistical Analyses

SV/CO data was analyzed *post-hoc* by exporting cvs.-files generated by the PhysioFlow^®^ software (Manatec Biomedical, Folschviller, France) to SPSS 27.0 for Mac (IBM Corporation, Armonk, NY). SV and CO were corrected for body surface area, yielding SV index (SVI) and CI that were used in the analyses. Descriptive statistics are reported as median with interquartile range (IQR). Pearson correlation coefficient (*r*) was calculated for CI and SVI measured by PhysioFlow^®^, and HR, mean arterial BP and blood lactate as indirect measures of systemic perfusion. We analyzed pooled data from all piglets 30 min from initiation of asphyxia.

## Results

The median (IQR) weight of the asphyxiated piglets was 2,045 (1,950–2,145) g. According to protocol, the piglets were euthanized or died at asystole (no resuscitation/no ROSC) (*n* = 11), 30 min after ROSC (*n* = 1), or 9.5 h after ROSC (*n* = 3).

Baseline characteristics, and characteristics of asphyxia and resuscitation are presented in Table 1.

**Table 1:** Characteristics of 15 asphyxiated piglets monitored with PhysioFlow^®^ signal-morphology impedance cardiography.

	**Variable**	**Median (IQR), unless otherwise specified**
Baseline	Sex [female number (%)]	10 (67)
	Weight (g)	2,045 (1,950–2,145)
	Baseline hemoglobin (g/dL)	7.3 (7.0–7.6)
	Heart rate (beats per minute)	172 (154–184)
	Mean arterial blood pressure (mm Hg)	73 (58–74)
	Baseline lactate (mmol/L)	1.6 (1.1–2.0)
Asphyxia, asystole and cardiopulmonary resuscitation	Lactate at asystole (mmol/L)	15.0 (9.1–17.0)
	Duration of asphyxia (min)	30 (20–35)
	Duration of cardiopulmonary resuscitation (sec) *n* = 4	76 (60–201)
	Number of adrenaline doses	4 (2–4)
	Stroke volume index (mL/m^2^)^*^	25.6 (11.5–38.9)
	Cardiac index (L/m^2^/min)^*^	4.3 (2.0–5.8)

*IQR, interquartile range*.

* *Summary measure for all piglet 30 min from initiation of asphyxia, cardiopulmonary resuscitation and post-resuscitation observation*.

Median (IQR) time monitored with the PhysioFlow^®^ for individual piglets was 106 (68–150) min, and the number of registrations per piglet 619 (233–971), range 92–6,759.

### Technical Issues

As can be seen from Figure 1, the blue Ambu^®^blue electrodes are large for an individual with a body surface area of 0.14 m^2^. Alternative electrodes [e.g., 3M™ Red Dot™ ECG Monitoring Electrodes (3M Saint Paul, Minnesota, United States)] were tested. However, in addition to consistently large sized electrode patches, these alternatives did not provide us with the same signal quality as the Ambu^®^blue electrodes. The ICG signal was obtained from start of asphyxia, but lost during cardiopulmonary resuscitation.

### Data Quality Assessment

**Figure 3:**
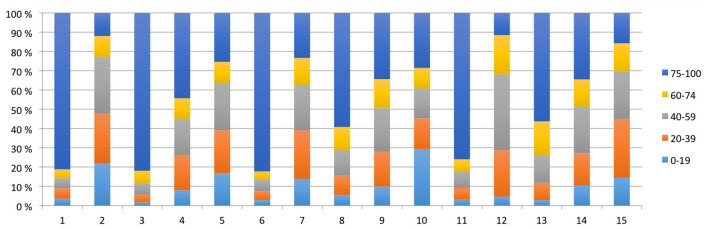
All measurements of each piglet are represented by bars and subdivided by signal quality (%). The total number of measurements for each piglet equals 100%.

Characteristics of the registrations in individual piglet are presented in Table 2 and Figure 3. The PhysioFlow^®^ signal quality varied throughout individual experiments, and in some piglets, the signal was completely lost during brief periods in addition to during cardiopulmonary resuscitation. The piglets with the longest observation periods (# 3 and # 6, Figure 3) had the highest proportion of good quality measurements. We included a total of 10.148 (48.3%) out of 20.991 registrations during and after asphyxiation and cardiopulmonary resuscitation. Analyses were performed on 1.254 registrations collected 30 min from initiation of asphyxia, i.e., in piglets with brief asphyxia times, this time period included cardiopulmonary resuscitation and post-resuscitation observation.

**Table 2:** Characteristics of PhysioFlow^®^ signal-morphology impedance cardiography measurements in 15 asphyxiated piglets.

**Piglet #**	**Euthanasia time**	**Signal quality (%) median (IQR)**	**Time registered (min)**	**Total number of measurements/registrations**	**Measurements with signal quality ≥ 75% [*n* (%)]**	**Measurements first 30 min of asphyxia and post-resuscitation observation (*n*)**
1	Asystole	100 (85–100)	114	971	122 (13)	86
2	Asystole	40 (20–55)	43	92	11 (12)	11
3	9.5 h	100 (85–100)	586	6,759	5,536 (82)	8
4	Asystole	65 (35–100)	150	877	311 (35)	286
5	Asystole	45 (25–75)	82	138	15 (11)	14
6	9.5 h	100 (95–100)	626	3,303	2,704 (82)	67
7	9.5 h	45 (25–70)	66	2,621	603 (23)	101
8	Asystole	85 (50–100)	126	781	134 (17)	127
9	Asystole	55 (35–85)	68	233	80 (34)	70
10	Asystole	45 (15–80)	134	806	71 (9)	68
11	Asystole	100 (75–100)	86	619	196 (32)	183
12	Asystole	50 (35–60)	106	453	37 (8)	12
13	30 min	75 (55–90)	330	2,899	210 (7)	149
14	Asystole	55 (35–90)	73	261	90 (34)	56
15	Asystole	40 (25–61)	61	178	28 (16)	16
**Total**			**2,808**	**20,991**	**10,148**	**1,254**

*Euthanasia is the practice of ending the life of an animal in a controlled fashion*.

*Signal quality (%) median (IQR)- Signal quality is given as a number in percentage where above 75% is good signal quality. The median (IQR) indicates median with interquartile range in parenthesis*.

*The rest of the bold values and their meaning is self-explanatory*.

### Physiological Results

**Figure 4:**
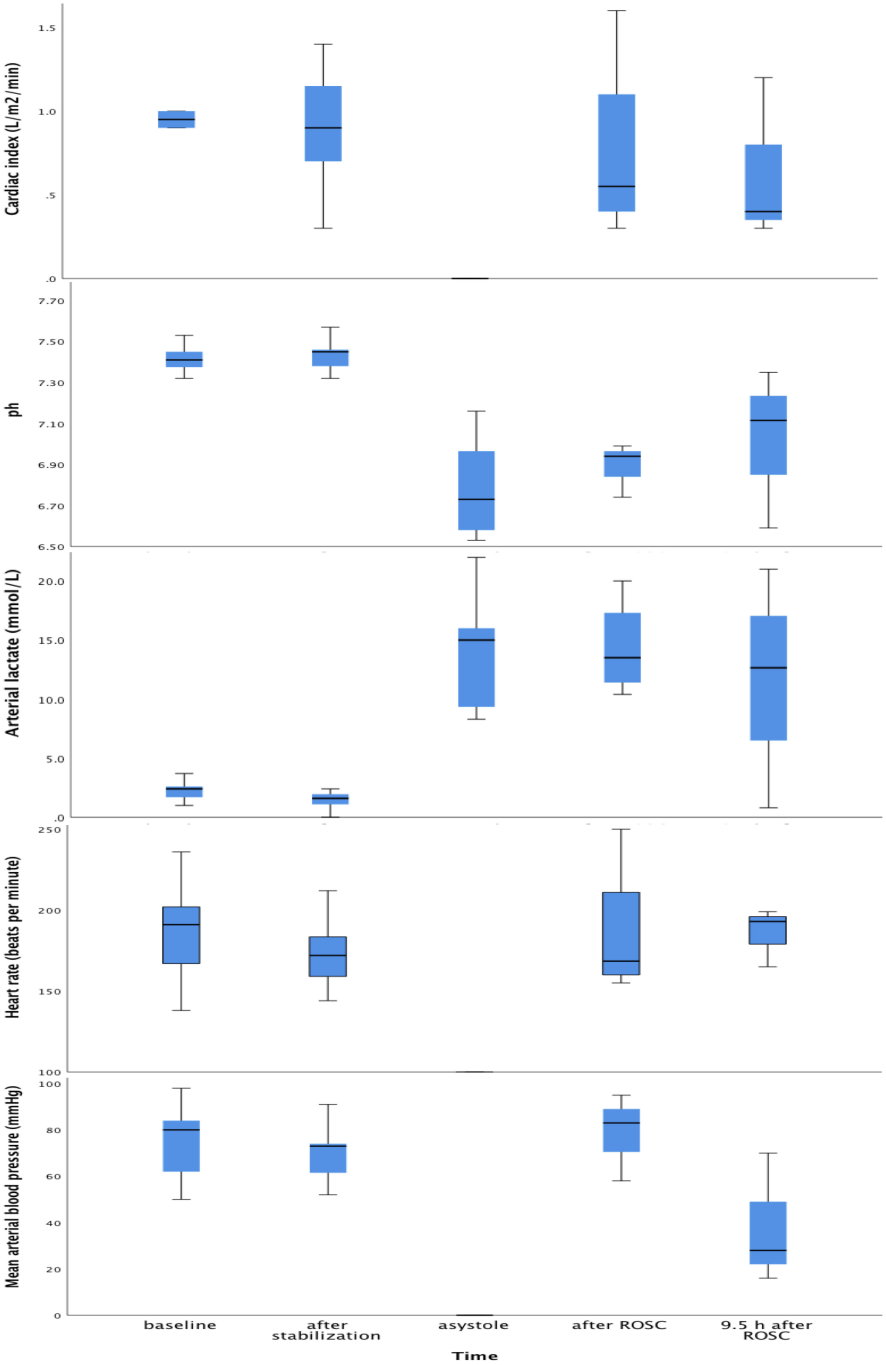
Box plots of cardiac index, pH, lactate, heart rate and arterial blood pressure at baseline, after post-surgical stabilization, at asystole and 9.5 h after return of spontaneous circulation. Within each box, the horizontal black line represents the median value; boxes extend from the 25th to the 75th percentile; while the whiskers represent the minimum and maximum values, respectively.

Figure 4 shows CI, pH, lactate, HR and mean arterial BP at equivalent time points.

There was a strong positive correlation between CI and SVI (*r* = 0.90, *p* < 0.001), and between CI and HR (*r* = 0.446, *p* < 0.001).

There was no correlation between CI and mean arterial BP (*r* = 0.251, *p* = 0.51), or between CI and lactate (*r* = −0.010, *p* = 0.98).

There was no correlation between SVI and mean arterial BP (*r* = 0.100, *p* = 0.80), or between SVI and lactate (*r* = −0.048, *p* = 0.90).

There was a negative, but not significant correlation between lactate and mean arterial BP (*r* = −0.586, *p* = 0.097).

## Discussion

In asphyxiated neonatal piglets with a body surface area of 0.14 m^2^, the PhysioFlow^®^ SM-ICG™ signal quality was acceptable in half of the registrations. However, the fraction of good quality registrations varied substantially between individual piglets. Our quality assessment results are better than those of a delivery room study using EV ICG where ¾ of measurement were excluded due to poor signal quality (22). However, caution should be taken when comparing the results of different ICG devices provided by different manufacturers, as they are not interchangeable. The Physioflow^®^ is largely based on traditional ICG, but with the addition of “Signal Morphology” technology which claims to make Physioflow^®^ more stable and reliable. SM-ICG bears the closest resemblance to EV (in contrast to BR), but with different methods for quantifying thoracic electrical bioimpedance.

The mean (standard deviation) CI measured by PhysioFlow^®^ in a pediatric study (18) was 3.17 (0.65) l/m^2^/min. Our CI-data were comparable, taking into account the hyperdynamic post-asphyxial state of the animals demonstrated in our previous piglet asphyxia study using echocardiography (23). The lack of correlation between CI, and BP and lactate, is in agreement with other studies (2).

Hemodynamically significant intra- and extra-cardiac shunts are likely to affect CO measured by ICG. Fugelseth et al. (24) showed in a study of 20 asphyxiated newborn piglets that two piglets (10%) had a patent ductus arteriosus at baseline. Asphyxia may induce reopening of the ductus arteriosus, and two of the initially closed ducts reopened during asphyxia (24). All ductal shunts were left to right. Most ICG devices assume left ventricular dominant circulations, which may be another source of error in newborn infants. Depending on the degree and the direction of intra- and extracardial shunts, this can either lead to an underestimate (right-to-left) or an overestimate (left-to-right) of systemic blood flow when estimating left ventricular CO (25).

The PhysioFlow^®^ has performed well in adult studies (14, 15). However, in the pediatric study by Taylor et al. (18), PhysioFlow^®^ failed to measure CO in two patients, likely due to contact failure of electrodes. Physiologically implausible data were defined by the authors as HR < 40 beats per minute, SV <5 ml, and CI < 0.7 l/m^2^/min. Due to the extreme conditions of our asphyxia protocol, we could not apply such exclusion criteria to our data. However, we did exclude registrations with a signal quality < 75%.

Our results are only partly in agreement with previous pediatric PhysioFlow^®^ data that indicate that measurement in small individuals is not feasible. Considering that low and high CO situations have been found to be associated with reduced accuracy compared to “normal” output ranges (26), a good signal quality in 50% of registrations in our SM-ICG study may be a reasonable point of departure for further optimizing this particular ICG technology. Low or high output states are those that may be the most relevant to monitor. PhysioFlow^®^ has a modified proprietary algorithm, which avoids calculation of baseline impedance, historically associated with errors and instability. According to the manufacturer (Timik AS, Norway, personal communication) new program ware and firmware have been developed which will improve performance and signal processing even further.

A limitation of our study was that we did not measure CO/CI by other means than ICG. In the pediatric study, PhysioFlow^®^- and MRI-CI were within 20% of each other in 8/23 (35%, 95% confidence interval: 19–55%) and within 30% of each other in 14/23 patients (61%, 95% confidence interval: 41–78%). CI was over- and underestimated by ≥20% in about 1/3 of patients each. A lower average CI and single ventricle physiology were associated with negative bias, whereas congenital heart disease (vs. normal heart) was associated with a positive bias.

Some authors have pointed out that most of the routinely used CO measuring devices have significant error themselves. E.g., the error of thermodilution is 13% despite averaging of triplicate measurements in an adult critical care population. Echocardiography has a precision of around 30% compared with thermodilution (27). O'Neill et al. (28) performed a review and concluded that normative data or intervention thresholds from echocardiography should not be used in the interpretation of ICG-derived SV and CO. Comparisons with unreliable “gold standards” may confuse validation testing of new technology. Disregarding this, EV has been shown to have similar precision as echocardiography in preterm and term newborns with little bias when compared to echocardiography, given an EV-signal quality index ≥ 80% (12, 29), as well as in infants being treated with therapeutic hypothermia (30).

As previously mentioned, our results are superior to a delivery room study using EV ICG where ¾ of measurement had to be excluded due to poor signal quality (22). However, in contrast to a clinical delivery room study, our CO/CI measurements were performed in sedated animals under strictly controlled laboratory conditions, eliminating motion artifacts and other noise. On the other hand, Slagt et al. (31) were able to acquire good quality EV-measurements during patient helicopter transport, showing that movement does not have to be a limiting factor after all. The high number of registrations represents the main strength of our study and increases the robustness of our results.

## Conclusions

Researchers have claimed that, if taking into account the known limitations of non-invasive hemodynamic measurement methods, they could be used in clinical care (32). Our data support utility of the PhysioFlow^®^ for trend monitoring in asphyxia, but further refining electrode size and placement, program ware and firmware, may be necessary before taking the PhysioFlow^®^ to the clinical setting with compromised newborn infants. Further research investigating factors influencing inter-individual variations in signal quality should be explored.

## Data Availability Statement

The raw data supporting the conclusions of this article will be made available by the authors, without undue reservation.

## Ethics Statement

The animal study was reviewed and approved by the Norwegian Council for Animal Research.

## Author Contributions

GB and AS performed data analysis and interpretation, and drafted the initial version of the manuscript. RS was involved in conceptualizing, designing and conducting the study, and CK in the conceptualizing, data analysis and interpretation. All authors participated in critical revision of the manuscript for important intellectual content, approved the final manuscript as submitted, and agree to be accountable for all aspects of the work.

## Funding

GB has received a grant from the Laerdal Foundation of Acute Medicine.

## Conflict of Interest

The authors declare that the research was conducted in the absence of any commercial or financial relationships that could be construed as a potential conflict of interest.

## Publisher's Note

All claims expressed in this article are solely those of the authors and do not necessarily represent those of their affiliated organizations, or those of the publisher, the editors and the reviewers. Any product that may be evaluated in this article, or claim that may be made by its manufacturer, is not guaranteed or endorsed by the publisher.

## Abbreviations

HR: heart rate
BP: blood pressure
CO: cardiac output
SV: stroke volume
ICG: impedance cardiography
SM-ICG: Signal-morphology impedance cardiography
CI: cardiac index
SVI: stroke volume index
IQR: interquartile range.
